# Diagnostic and therapeutic value of P2Y12R in epilepsy

**DOI:** 10.3389/fphar.2023.1179028

**Published:** 2023-05-10

**Authors:** Xiang Chen, Qi Wang, Jie Yang, Li Zhang, Ting-Ting Liu, Jun Liu, Bin-Lu Deng, Jie Liu

**Affiliations:** ^1^ Department of Neurology, School of Clinical Medicine, Southwest Medical University, Luzhou, China; ^2^ Department of Neurology, Sichuan Academy of Medical Sciences, Sichuan Provincial People’s Hospital, Chengdu, China; ^3^ Zunyi Medical University, Zunyi, China; ^4^ Electrophysiology Unit, Department of Neurology, Chengdu Fourth People’s Hospital, Chengdu, China; ^5^ Department of Neurology, Sichuan Provincial People’s Hospital, University of Electronic Science and Technology of China, Chengdu, China; ^6^ Department of Geriatric Neurology, Qinglongchang Ward, Chengdu Sixth People’s Hospital, Chengdu, China

**Keywords:** P2Y12 receptor, epilepsy, seizures, neuroinflammation, diagnosis, therapy

## Abstract

There lacks biomarkers in current epilepsy diagnosis, and epilepsy is thus exposed to inadequate treatment, making it necessarily important to conduct search on new biomarkers and drug targets. The P2Y12 receptor is primarily expressed on microglia in the central nervous system, and acts as intrinsic immune cells in the central nervous system mediating neuroinflammation. In previous studies, P2Y12R in epilepsy has been found capable of controlling neuroinflammation and regulating neurogenesis as well as immature neuronal projections, and its expression is altered. P2Y12R is involved in microglia inhibition of neuronal activity and timely termination of seizures in acute seizures. In status epilepticus, the failure of P2Y12R in the process of “brake buffering” may not terminate the neuronal hyperexcitability timely. In chronic epilepsy, neuroinflammation causes seizures, which can in turn induce neuroinflammation, while on the other hand, neuroinflammation leads to neurogenesis, thereby causing abnormal neuronal discharges that give rise to seizures. In this case, targeting P2Y12R may be a novel strategy for the treatment of epilepsy. The detection of P2Y12R and its expression changes can contribute to the diagnosis of epilepsy. Meanwhile, the P2Y12R single-nucleotide polymorphism is associated with epilepsy susceptibility and endowed with the potential to individualize epilepsy diagnosis. To this end, functions of P2Y12R in the central nervous system were hereby reviewed, the effects of P2Y12R in epilepsy were explored, and the potential of P2Y12R in the diagnosis and treatment of epilepsy was further demonstrated.

## 1 Introduction

Epilepsy is one of the most common and disabling chronic neurological disorders, which affects over 70 million people globally and imposes considerable socio-economic burdens (Beghi, 2020; Hauser, 2019; Thijs et al., 2019). Besides, it is defined as a chronic disorder of spontaneous seizures caused by an imbalance between excitability and inhibition in the brain, and the neurobiological, cognitive, psychological and social consequences of the condition (Mo et al., 2019; Sarmast et al., 2020). Epilepsy can be caused by brain dysplasia, brain injury (infections, stroke, tumors, and traumatic brain injury (TBI)), also genetic abnormalities (e.g., genetic polymorphisms, copy number variants, or *de novo* mutations) (Rees, 2010; Pitkänen, A., and Lukasiuk, 2011; Pitkanen and Engel, 2014; Klein et al., 2018). The pathological process of epilepsy includes synaptic reorganization, blood-brain barrier (BBB) disruption, alterations in neurotransmitter release, abnormal neurogenesis, neurodegeneration, and neuroinflammation (Jacobson and Boeynaems, 2010; Henshall and Kobow, 2015; Pitkanen et al., 2015; Guzman and Gerevich, 2016). At present, the diagnosis of epilepsy remains complex and clinically challenging. Video electroencephalography (EEG) monitoring is still the gold standard in hospitals, but this method is time-consuming, costly and low-yielding, and requires a high level of expertise (Engel and Pitkanen, 2020). The clinical manifestations of epilepsy are diversified, and some epilepsies (especially non-convulsive epilepsies) are easily confused with other disorders and may be misdiagnosed or missed, thus causing incorrect or unnecessary treatment. As a result, the diagnosis of epilepsy remains inadequate. Biomarkers have potential in the diagnosis of epilepsy (Engel and Pitkanen, 2020), which are useful in the diagnosis, differential diagnosis and prediction of seizures, and also have potential therapeutic uses, such as identifying persistent seizures and their mechanisms, predicting the effectiveness of antiepileptic drugs, assessing the likelihood of recurrence after stopping treatment, as well as assessing susceptibility to drug side effects (Sueri et al., 2018; Engel and Pitkanen, 2020; Perucca, 2021), making it necessarily important to search for biomarkers of epilepsy and further improve the diagnostic accuracy of epilepsy.

The current treatment choice for epilepsy is still antiepileptic drugs, with more than 30 antiseizure medications (ASMs) clinically available (Perucca, 2021). However, antiepileptic drugs control only 70% of patients with epilepsy, have no appreciable effect on disease process, and can cause serious side effects (such as fatigue, irritability, and dizziness) (Bialer and White, 2010; Thijs et al., 2019). Besides medication, additional treatments include neuromodulation devices and stimulators (such as vagus nerve stimulators (VNS)), reactive neuromodulation (RNS), Deep Brain Stimulator (DBS), resective epilepsy surgery and ketogenic diet (Rincon et al., 2021; Dyńka et al., 2022; Foutz and Wong, 2022; Hartnett et al., 2022; Imdad et al., 2022; Rho and Boison, 2022). In this case, it is still necessary to search for alternative therapeutic schemes with new mechanism to modify the disease progression and provide effective treatment for drug-resistant epilepsy (Alves et al., 2018). Therefore, different mechanisms of therapeutic regimens should be currently developed to suppress epilepsy and even to influence epilepsy progression.

For note, there is growing academic interest in the effect of neuroinflammation in epilepsy (Terrone et al., 2017). Microglia are intrinsic immune cells of the central nervous system that mediate neuroinflammation, on which, P2Y12 receptor (P2Y12R) is mainly expressed in the central nervous system (CNS); Thus, P2Y12R plays an influential pathophysiological role in the neuroinflammatory response to epilepsy and is potentially valuable for the diagnosis and treatment of epilepsy. This review will focus on the P2Y12 receptor, giving a brief overview of the structure and expression of P2Y12R, detailing the effects of P2Y12R in epilepsy, particularly neuroinflammation, and highlighting potential applications of P2Y12R in the diagnosis and treatment of epilepsy.

## 2 Potential therapeutic value of P2Y12R

P2Y12R is a member of the purinergic signaling family (Dahlquist and Diamant, 1974; Hynie, 1995; Burnstock, 2004; Burnstock, 2007). Purinergic signaling, which includes nucleotides (e.g., adenosine triphosphate (ATP)), their hydrolysis products (adenosine diphosphate (ADP), adenosine monophosphate (AMP)), nucleosides (e.g., adenosine), enzymes (CD39, CD73) and purinergic *p*) receptors, was proposed by Burnstock in 1972 and has been recognized as a new etiological factor or promising potential target for the treatment of central nervous system disorders (Huang et al., 2021; Li et al., 2022; Trinh et al., 2022; Iring et al., 2021; Ribeiro et al., 2022). The binding of extracellular nucleosides and nucleosides to purinergic receptors leads to the activation of intracellular signaling pathways, which in turn gives rise to changes in cell physiology (Zimmermann, 2006; Burnstock, 2008; Burnstock, 2018; Burnstock, 2020). ATP and its derivatives, diadenine nucleotides, act as partial agonists or antagonists of P2Y12R (Kauffenstein et al., 2004), where ADP is an endogenous agonist of P2Y12R (Bodor et al., 2003). P2Y12R, coupled with Gi protein, inhibits adenylate cyclase, thus reducing the production of cAMP and affecting intracellular calcium concentration (Cheffer et al., 2017). The crystal structure of P2Y12R is composed of seven hydrophobic transmembrane regions (TMs), which are connected by three extracellular loops (ELs) and three intracellular loops (Zhang K et al., 2014; Zhang et al., 2015). P2Y12R contains 342 amino acid residues and has two potential N-linked glycosylation sites at its extracellular amino terminus, which regulates its activity (Takasaki et al., 2001; Cattaneo, 2015). P2Y12R has four cysteine residues at the ELs (Cys 17, 97, 175, 270), which form two disulfide bonds that act accordingly upon stimulation/inhibition (Algaier et al., 2008; Ding et al., 2009; Hillmann et al., 2009; Gomez Morillas et al., 2021). Initially, the P2Y12 receptor was thought to be expressed only on platelets (Nie et al., 2017), and drugs that block P2Y12R (e.g., clopidogrel) were widely used as antiplatelet aggregation agents for the treatment of cardiovascular diseases (Raju et al., 2008). However, Hollopeter et al. found that P2Y12R was expressed in the brain (Hollopeter et al., 2001), and other studies further showed that it was also expressed on microglia, vascular smooth muscle cells, dendritic cells, lymphocytes, brown adipocytes, osteoblasts, osteoclasts, and primary cilia of bile duct cells (Wihlborg et al., 2004; Ben Addi et al., 2010; Diehl et al., 2010; Kronlage et al., 2010; Rauch et al., 2010; Gachet, 2012; Liverani et al., 2014; Jacobson et al., 2020; Mansour et al., 2020). It is currently believed that in the central nervous system (CNS), P2Y12R is primarily expressed in microglia and is stably expressed during the development of human brain (Butovsky et al., 2014; Zhang, Y et al., 2014; Lou et al., 2016; Cserép et al., 2020). Microglia are the first cells to respond to brain injury and neurodegeneration. Therefore, P2Y12R is considered as a marker to distinguish microglia cells from other brain cells and myeloid cells throughout human life (Moore et al., 2015; Mildner et al., 2017; Hammond et al., 2018; Cserép et al., 2020).

In the SE mouse model induced intra-amygdala KA and intraperitoneal pilocarpine, the transcription levels of uracil-sensitive P2Y receptors (P2Y2R, P2Y4R, and P2Y6R) in the hippocampus were increased, while those of adenine-sensitive P2Y receptors (P2Y1R, P2Y12R, and P2Y13R) were decreased. However, at the protein level, the expression of P2Y1, P2Y2, P2Y4 and P2Y6 was increased, while that of P2Y12 was decreased after SE, which might be attributed to the increased G protein coupling of the receptor to the P2Y receptor coupled to Gq, as well as the downregulated or unchanged P2Y receptor coupled to Gi (Alves et al., 2017). Additionally, in chronic epilepsy, levels of P2Y1R, P2Y2R, and P2Y6R transcripts and P2Y1, P2Y2, and P2Y12 proteins were elevated, while those of other P2Y receptors were unchanged (Alves et al., 2017).

P2Y12R is essential for maintaining brain homeostasis. A recent study shows that P2Y12R deficiency disrupts neuronal precursor cell proliferation and leads to structural abnormalities in developmental and adult cortical cells (Cserép et al., 2022). Meanwhile, microglia affect neuronal proliferation in a P2Y12R-dependent manner and regulate neurogenesis and projections of immature neurons (Mo et al., 2019; Cserép et al., 2022). Besides, P2Y12R also promotes the proliferation of adult mouse subventricular zone (SVZ) cells (Suyama et al., 2012). Interestingly, the activation of P2Y1R and P2Y12R induces astrocyte proliferation *in vitro* (Quintas et al., 2011). Furthermore, in astrocyte and microglia co-cultures, microglia P2Y12R and P2Y13R are involved in blocking ADPβS-mediated astrocyte proliferation (Quintas et al., 2014). P2Y12R and P2Y13R are integrally linked, with the latter enhancing the chemotactic response of the former (Kyrargyri et al., 2020). Also, microglia P2Y12 is required for synaptic plasticity in the mouse visual cortex, and the destruction of P2Y12 reduces the branching of basal microglia processes under homeostasis, indicating a close correlation between microglia branching and P2Y12 expression (Haynes er al., 2006; Sipe et al., 2016). In addition, microglia-neuron interaction is known as microglial protuberance convergence (MPC) toward neuronal axons and dendrites (Eyo et al., 2018). Cserép et al. observed the unique nanostructure known as somatic purinergic connections of microglia-neuron connections in mouse and human brains, and claimed that somatic preferences in the adult brain promote the site formation of Kv2.1 clusters in neuronal cytosol, which are associated with neuronal mitochondrial activity, and are highly dynamic and P2Y12R-dependent (Cserép et al., 2020; Cserép et al., 2022). Microglia P2Y12Rs defines the somatic purinergic connection on doublecortin-positive (DCX+) developing neurons that enable microglia to monitor and shape neural development and facilitate neuronal integration within cortical networks (Cserép et al., 2022). In capillary injury, P2RY12-mediated activation of paravalvular microglia and microglia protrusions rapidly forms dense plexiform aggregate at the site of injury, which consists of membrane attachments expressing E-calmodulin, and acts as a physical barrier that temporarily closes the blood-brain barrier (Lou et al., 2016).

Microglia participate in neuroinflammation as resident immune cells in the brain. Under physiological conditions, microglia take a branching form, extending and retracting in all directions to survey the brain (Gomez Morillas et al., 2021). Purinergic signaling will be the primary system for triggering microglial cell extension (Koizumi et al., 2013), and microglia protrusions in P2Y12R-deficient mice do not extend, suggesting the involvement of P2Y12R in microglia protrusion extension and tropism (Haynes et al., 2006). Besides, ATP/ADP also inhibits the adenylyl cyclase pathway downstream of P2Y12R through the induced activated phospholipase C (PLC) and phosphatidylinositol 3-kinase (PI3K) pathways, and the activation of integrin-β1 and its accumulation at the end of extended protrusions are involved in the extension of microglia protrusions in brain tissues (Ohsawa et al., 2010; Ohsawa and Kohsaka, 2011). In addition, the activation of P2Y12R has been reported to enhance the activity of the twik-related halothane inhibitory K+ channel (THIK-1), which regulates microglia differentiation and brain monitoring functions under physiological conditions and maintains the “resting” potential of microglia (Madry et al., 2018; Gomez Morillas et al., 2021). In this case, P2Y12R is considered to mediate the monitoring role of microglia in the resting state.

### 2.1 Antiepileptic effect of P2Y12R works by controlling neuroinflammation

Neuroinflammation is an essential pathological process in epileptogenesis, which gives rise to the hyperexcitability of the brain (Engel et al., 2021). Currently, the potential applications of targeting inflammatory cytokines and purinergic receptors in epilepsy have been fully demonstrated (Burnstock, 2017; Alves et al., 2018; Rana and Musto, 2018). The Anne Schaefer team found that microglia inhibit neuronal activity in a negative feedbacking way similar to that of inhibitory neurons, acting as a “brake buffer” (Badimon et al., 2020), and matter considerably in the process of inhibiting neuronal activity in P2Y12R. Neuroinflammation overexcites neurons, and seizures result (Vezzani et al., 2010). During a seizure, ATP is released from the cell and metabolic ADP activates P2Y12R, which modulates the microglial phenotype after a seizure. ATP via P2Y12R attracts microglia, and the microglia ATP/ADP then hydrolyzes ectoenzyme CD39 into AMP. AMP is converted by CD73 into adenosine, which mediates the inhibition of neuronal activity via adenosine receptor A1R (Badimon et al., 2020), and finally acute epilepsy ceases. In status epilepticus, the failure of P2Y12R in the process of “brake buffering” may not terminate the neuronal hyperexcitability in time. Models of status epilepticus have been used in previous experiments, and the exacerbation of seizures in P2Y12R knockout mice again supports this idea (Eyo et al., 2014). In chronic epilepsy, neuroinflammation causes seizures on the one hand, which can in turn induce neuroinflammation, while on the other, neuroinflammation can lead to neurogenesis, which in turn causes abnormal neuronal discharges that lead to seizures. Recurrent seizures perpetuate chronic inflammation, which may be the cause of recurrent chronic epilepsy.

P2Y12R may be a drug target in epilepsy, where there is growing evidence proving its role in regulating neuroinflammation. Besides, an interaction exists between neuroinflammation and epilepsy. Previous studies have shown that neuroinflammation can cause epilepsy, seizures can also result from it, and neuroinflammation can promote neuronal hyperexcitability and lead to seizures (Vezzani et al., 2010). The mRNAs encoding P2Y12 and P2Y13 receptors are observed in the rat brainstem, where there are also cell bodies of catecholaminergic neurons innervating the hippocampus, and the activation of P2Y12R inhibits the release of norepinephrine in the hippocampus to affect neuronal excitability (Csölle et al., 2008). A variety of inflammatory mediators can be detected in brain tissue sections from epilepsy patients after surgical resection (Vezzani and Granata, 2005; Choi et al., 2009). Animal experiments have also indicated that extracellular ADP enhances microglia inflammation by acting on P2Y12R to activate nuclear factor-κ-gene binding (NF-κB) and NOD-like receptor thermal protein domain associated protein 3(NLRP3) inflammatory vesicles and the release of IL-1β and IL-6, thereby increasing seizures (Cieślak et al., 2017; Suzuki et al., 2020). Besides, IL-1β induces seizures through the activation of the GluN2B subunit of the NMDA receptor, and increases the production of GluN2B mRNA and the upregulation of NMDA receptors on postsynaptic cells. In addition, 24 h after pentylenetetrazole (PTZ)-induced status epilepticus, it was observed in the rat hippocampal slices that GluN2B subunit expression is increased and LTP at hippocampal synapses is reduced, leading to impaired synaptic plasticity (Viviani et al., 2003; Postnikova et al., 2017). There are also alterations in the concentration of IL-1β in TLE that reduce GABA-mediated neurotransmission up to 30%, and lead to seizures due to neuronal hyperexcitability (Roseti et al., 2015). Both IL-1β and IL-6 reduce long-term potentiation (LTP), and microglia stimulation and elevated IL-1β levels will also result in the upregulation of IL-6 (Erta et al., 2012; Gruol, 2015; Rana and Musto, 2018). Upregulation of IL-6, an inflammatory cytokine, reduces LTP and hippocampal neurogenesis, while changes in hippocampal structure and morphology as well as the hyperexcitability of the hippocampal region lead to epileptogenesis (Samuelsson, 2005; Erta et al., 2012; Pineda et al., 2013; Levin and Godukhin, 2017). Lipopolysaccharide (LPS) that induces neuroinflammation may also lower the threshold for seizures (Sayyah et al., 2003; Heida and Pittman, 2005; Galic et al., 2008; Auvin et al., 2009; Auvin et al., 2010). Additionally, seizure activity itself can induce inflammation in the brain, and recurrent seizures can perpetuate chronic inflammation (Vezzani et al., 2010). These inflammatory factors can influence the severity and recurrence of seizures, thereby forming a vicious cycle (Vezzani et al., 2010). However, the role of P2Y12R in it is still not clear at present, and needs further explorations.

Under neuroinflammatory conditions, ATP is released extracellularly, activates both the P2Y12R receptor as ATP/ADP, and stimulates the adenosine receptor (AR) A3 as a metabolic receptor. P2Y12R activation acts as the first responder to microglial activation, and acts synergistically with A3. Meanwhile, high expression of both mediates the extension of microglial protrusions towards the injury site. Subsequently, upregulation of ARA2A and downregulation of P2Y12R induce microglia protrusion retraction. The microglia migratory activity is controlled by the interaction of P2Y12R and P2X4R. When the protrusions are fully retracted, microglia will transform into amoeboid morphology. Finally, P2Y6R and P2X4R activation induces phagocytosis and pinocytosis, respectively, while P2X4R and P2X7R are involved in secretory activity (Rivera et al., 2016; Fekete et al., 2018; Illes et al., 2020; Gomez Morillas et al., 2021). The activation of a number of other P1 and P2 receptors (Koizumi et al., 2013) regulates P2Y12 receptor-mediated responses, suggesting a close interaction between P2Y12R and other purinergic receptors that act together to get involved in neuroinflammation.

P2Y12 receptor-mediated greater response to the signals of “eat me” and “find me” renders activated microglia the ability to intervene more rapidly in damaged cells (Avignone et al., 2008). P2Y12R is involved in membrane fluffing, protrusion extension or retraction, and chemotactic motions of microglia. Activation of P2Y12R induces microglia reactivity and causes microglia protrusion growth, which also mediates microglia chemotaxis via phosphatidylinositol 3-kinase (PI3K)/phospholipase C (PLC) signaling. This matters considerably for the clearance of infected cells or cellular debris and tissue recovery (Ohsawa et al., 2007; Irino et al., 2008; Engel et al., 2021). Similar to synaptic extension, P2Y12 receptor-mediated migration of microglia also involves inhibition of the adenylate cyclase pathway and reduction of the cAMP levels and protein kinase A (Nasu-Tada et al., 2005). Charolidi et al. found that blocking microglia P2Y12 receptors with PSB0739 inhibits the release of chemokines (CCL2 and CXCL1), and microglia P2Y12R thus regulates the release of chemokines CCL2 and CXCL1 (Charolidi et al., 2015). All these processes depend on dual-pore domain-type potassium channels and are associated with alterations in mitochondrial membrane potential (Suzuki et al., 2020). Besides, microglia couple phagocytosis to apoptotic processes through P2Y12R signaling during development (Blume et al., 2020). The P2Y12R signaling pathway is involved in phagocytosis-mediated chemotaxis to the “find-me” signal ADP (Haynes et al., 2006), which is necessarily important for the rapid and efficient clearance of microglia-mediated apoptotic cells (Blume et al., 2020). Impaired microglial phagocytosis and reduced neurogenesis are observed in P2Y12 KO mice, and the involvement of microglia phagocytosis in a feedback loop that maintains homeostasis of adult hippocampal neurogenesis is also hereby revealed (Diaz-Aparicio et al., 2020).

Expression of P2Y12R on microglia depends on different activation patterns and CNS microenvironments (Colonna and Butovsky, 2017). Once activated, microglia are often categorized as the “classical” proinflammatory phenotype (M1) or the “alternative” anti-inflammatory type (M2) (Colonna and Butovsky, 2017). Under selective activation conditions (M2; IL-4 and IL-13), P2Y12 receptor expression increases, mediates microglia migration, and contributes to triggering an acute proinflammatory response to danger-related molecules released during central nervous system injury (Moore et al., 2015). However, under pro-inflammatory conditions, P2Y12R expression is reduced in rodent microglia, resulting in the failure to migrate up the ADP gradient (De Simone et al., 2010). In addition, P2RY12 expression is upregulated on the microglia of resting and non-inflammatory phenotype (M2), but downregulated during the M1/M2 transition of the post-activation inflammatory phenotype (Honda et al., 2001; Haynes et al., 2006). In this case, P2Y12R is considered a valuable sign that indicates the shift of the microglia functional pattern from chemotaxis to phagocytosis (Koizumi et al., 2013; Gomez Morillas et al., 2021), identifies non-pathological microglia, and differentiates activated microglia from stationary microglia (Mildner et al., 2017).

P2Y12R has been shown to regulate the microglia phenotype after seizures (Eyo et al., 2014; Eyo et al., 2017; Eyo et al., 2018). In the hippocampus of P2Y12 knockout (KO) mice intraperitoneally injected with kainic acid (KA) (18 mg/kg), the number of microglia primary protrusions is reduced, and seizures are worsened (Eyo et al., 2014). During seizures, neurons are highly active, and release glutamate from presynaptic terminals. Meanwhile, elevated glutamate levels activate neuronal NMDA receptors, thus causing an inward flow of extracellular calcium ions, while elevated intracellular calcium may lead to the release of ATP through ion channels such as pannexin 1 (PX1) or prepackaged vesicles. Besides, the released ATP diffuses into the extracellular space, forming a chemotactic gradient that activates P2Y12R in microglia, and inducing the extension of microglia protrusions toward neurons (Eyo et al., 2014). Acute seizures significantly alter the morphology and number of microglia in the hippocampal region, while glutamate treatment and prolongation of microglia protrusions during seizures play a neuroprotective role (Eyo et al., 2014). During seizures, P2Y12 receptors also regulate the microglial cell landscape through a cell shift mechanism (Eyo et al., 2018). In tissue slices from epileptic patients stained with fluorescent lectin, activation of the P2Y12 receptor initiates the extension of microglia protrusions (Haynes et al., 2006), which is similar to the situation in rodents, but the difference is that microglia retraction is triggered by the joint activation of P2Y1/P2Y13 receptors. Additionally, it was observed that low doses (1–10 μM for 15–30 min) of ADP induce the process extension for both initially amoeboid and ramified microglia, while high doses (1–2 mM for 30 min) of ADP cause process retraction and membrane ruffling (Milior et al., 2020). In summary, P2Y12R exerts a potential protective effect on epilepsy.

### 2.2 P2Y12R regulating neurogenesis and immature neuronal projections after seizures

Mo et al. ever claimed that P2Y12R regulates neurogenesis and immature neuronal projections after seizures in the intracerebroventricular kainic acid model, which composes some features of the epileptogenic environment (Danzer, 2018; Mo et al., 2019). Besides, it was hereby figured out that on the one hand, P2Y12R may promote the hyperactivity in epileptogenesis subsequent to the initial seizure, while on the other, as mentioned above, P2Y12R is involved in controlling neuroinflammation and microglia inhibiting neuronal activity and thus imposes an antiepileptic effect, so future work is needed to help further understand the detailed mechanism of the dual role of P2Y12R, epileptogenic and anti-epileptogenic (Mo et al., 2019). Interestingly, the removal of microglia exerts a more pronounced effect on seizure-induced neurogenesis than elimination of P2Y12R, suggesting that P2Y12R is not the only microglia protein that regulates seizure-induced neurogenesis (Mo et al., 2019). Therefore, other factors that regulate seizure-induced neurogenesis remain to be further investigated.

## 3 Potential diagnostic value of P2Y12R

### 3.1 P2Y12R expression during epilepsy

As described in the first study of expression in the P2YR family after seizures, increased P2Y12 Rtranscription in the hippocampus and increased P2Y12 activation-mediated microglial cell membrane currents 48 h after status epilepticus (SE) were observed in a mouse model of intraperitoneal kainic acid (KA)-induced SE (Avignone et al., 2008). The movement of microglia in the epileptic hippocampus toward P2Y12 receptor agonists was also found twice as rapid as in the normal hippocampus (Avignone et al., 2008). On the contrary, as stated before, in the SE mouse model induced intra-amygdala KA and intraperitoneal pilocarpine, P2Y12R transcription and protein expression were reduced after status epilepticus and P2Y12R protein levels were increased in chronic epilepsy (Alves et al., 2017). The expression of P2Y12R varied and diverged in different epilepsy models and at different stages of the disease. However, all indicated that P2Y12R expression was altered in epilepsy, which was endowed with the potential to assist in the diagnosis of epilepsy. The future application of positron emission tomography (PET) for the detection of P2Y12R itself in the brain needs further study.

### 3.2 Association between P2Y12R single-nucleotide polymorphism and epilepsy susceptibility

A study was reported on the association of single nucleotide polymorphisms (SNPs) in the P2Y12R gene with epilepsy, and it was found that carriers of the G allele of rs1491974 G>A or rs6798347 G>A may be associated with increased risk of epilepsy, with the rs1491974 G> A genotype and allele frequency differing significantly in females only, and individuals with this genotype may be exposed to more frequent seizures (Wang et al., 2022). However, a larger sample size and epilepsy classification are needed to further investigate the correlation between P2Y12R SNPs and epilepsy. P2Y12R SNPs currently have the potential to serve as a risk factor for epilepsy, with the potential for individualized diagnosis of epilepsy. There may be P2Y12R gene loci insensitive to P2Y12R-targeted drugs, and patients with insensitive loci can be removed by P2Y12R SNPs detection to achieve precision medicine in the future.

## 4 Conclusion and future perspectives

P2Y12R is closely associated with epilepsy and seizures (Table 1), and is thus endowed with potential application value in the diagnosis of epilepsy and seizures, which is also promising as an effective drug target. In acute seizures, P2Y12R is involved in the process of neuronal activity inhibition by microglia, terminating neuronal hyperexcitability and avoiding status epilepticus, while in chronic epilepsy, P2Y12R is a potential drug target by controlling neuroinflammation. Meanwhile, P2Y12R antagonists can inhibit neurogenesis and immature neuronal projections to prevent the next seizure, but the mechanism is still unclear and requires further exploration. The altered expression of P2Y12R in epilepsy and its own detection may support the diagnosis of epilepsy. In addition, P2Y12R SNPs can be a risk factor for epilepsy to inform the likelihood of epileptogenesis. In the future, it is expected that drugs targeting P2Y12R will act only on brain regions or cells where neurons are over-excited, terminate seizures, and delay seizure progression without affecting normal brain regions and cells. However, there are still considerable issues to be addressed before the application of P2Y12R to the diagnosis and treatment of epilepsy.

1) There are differences and divergences in the expression of P2Y12R in different epilepsy models and at different stages of pathogenesis. Changes in the expression and mechanism of P2Y12R across different seizure types, seizure severity and frequency, disease stages, animal models and epileptic patients should be additionally explored to provide new ideas for the diagnosis and treatment of epilepsy;
2) Changes in P2Y12 expression are not specific to epilepsy. A single change in purine signaling cannot be used as an independent diagnostic criterion, which, actually, needs to be combined with other measures for diagnostic evaluation in the clinical setting (Wong and Engel, 2022);
3) The association of P2Y12R SNPs with epilepsy requires a larger sample size of patients and different epilepsy types to verify the possibility of using P2Y12R as a genetic marker;
4) The current P2Y12R PET tracer for pro- and anti-inflammatory microglia has been validated in mouse models and human brain sections (Villa et al., 2018; Jackson et al., 2022; van der Wildt et al., 2022). Attempts should also be made to develop P2Y12R PET tracers and detect P2Y12R in animal models of epilepsy or in the human brain;
5) The dual mechanism of pro-epileptic and anti-epileptic action of P2Y12R in the whole process of epilepsy is still not completely understood, making it necessary to carry out further studies;
6) There is not yet a P2 receptor-based treatment that can entirely stop seizures, and P2 receptors are likely to be used for an adjunctive treatment (Engel et al., 2021). In early seizures, P2Y12R agonists inhibit neurohyperexcitability, control neuroinflammation, and assist in terminating seizures, while after seizures, P2Y12R antagonists inhibit neurogenesis and immature neuronal projections to prevent the next seizure. It is thus advisable for future research to combine P2Y12R with antiepileptic drugs to determine a more refined strategy for epilepsy treatment, particularly in refractory epilepsy.

**TABLE 1:** Summary of the effects of P2Y12R in epilepsy.

type		Seizure type	Methods/Models	Main results	References
Expression		SE	IP KA (18–22 mg/kg) mice	Increased P2Y12R transcription in the hippocampus.	Avignone et al. (2008)
		SE	intra-amygdala KA mice IP pilocarpine mice	Decreased P2Y12R transcription and protein expression after status epilepticus, and elevated levels of the P2Y12R protein in chronic epilepsy.	Alves et al. (2017)
Function	antiepileptic	SE	IP KA (18 mg/kg) P2Y12 KO mice	Increased seizure phenotype and reduced hippocampal microglial processes toard neurons in P2Y12-deficient mice.	Eyo et al. (2014)
		MTLE	Hippocampal tissue section stained with fluorescent lectin from patients	Low-dose ADP inducing microglial proboscis elongation by blocking P2Y12, while high-dose ADP causing process retraction and membrane fluffing by joint activation of P2Y1/P2Y13 receptors.	Milior et al. (2020)
	epileptogenic	seizure	ICV KA (0.032 mg/mL) mice	Microglial P2Y12 receptors regulating seizure-induced neurogenesis and immature neuronal projections.	Mo et al. (2019)
SNPs		epilepsy	patients with epilepsy	Possible correlation between the rs1491974 and rs6798347 polymorphisms of P2Y12R and epilepsy.	Wang et al. (2022)

**Abbreviations:** IP, intraperitoneal; ICV, intracerebroventricular; KA, kainic acid; KO, knockout; MTLE, mesial temporal lobe epilepsy; SE, status epilepticus; and SNPs, single-nucleotide polymorphisms.

In conclusion, much future research on the effect of P2Y12R in epilepsy should be carried out to further advance it into clinical practice.
